# Continuous Increase in Both Waiting and Process Time in the Emergency Rooms of Abruzzo, Italy

**DOI:** 10.3390/epidemiologia7030062

**Published:** 2026-05-04

**Authors:** Ippazio Cosimo Antonazzo, Camillo Odio, Giulia Chesini, Natalia Gregori, Sara Taormina, Enrico Zauli, Cecilia Acuti Martellucci, Maria Elena Flacco, Lamberto Manzoli

**Affiliations:** 1 Department of Environmental and Prevention Sciences, University of Ferrara, 44121 Ferrara, Italy; ippazio.antonazzo@unife.it (I.C.A.); mariaelena.flacco@unife.it (M.E.F.); 2 Healthcare Department, Abruzzo Region, 65127 Pescara, Italy; camillo.odio@regione.abruzzo.it; 3 Department of Medical and Surgical Sciences, University of Bologna, 40126 Bologna, Italy; giulia.chesini@studio.unibo.it (G.C.); natalia.gregori@studio.unibo.it (N.G.); sara.taormina@studio.unibo.it (S.T.); enrico.zauli@studio.unibo.it (E.Z.); c.acutimartellucci@unibo.it (C.A.M.)

**Keywords:** emergency department, emergency room, waiting time, length of stay: quality of healthcare services, Italy

## Abstract

Background/Objectives: A recent report by the Italian Ministry of Health showed that a high proportion of Emergency Department (ED) visits exceed the maximum recommended thresholds for both waiting time and overall length of stay. As no overall quantitative data are available on the magnitude, temporal evolution, and underlying drivers of ED performance from Southern Italian regions, we analyzed ED data from the Abruzzo region during the years 2017–2024, and evaluated potential predictors of prolonged waiting and process time. Methods: Official, administrative data from all the regional EDs were collected, and information on personnel, location and organizational change was obtained through a dedicated survey. All analyses were stratified and/or adjusted by triage code, hospital, age and sex. Results: From 2017 to 2024, a total of 3,563,565 accesses were recorded in the 16 regional EDs. From 2021, waiting time for the first visit steadily and progressively increased, reaching an average of 78 min in 2024 (+56.0%), largely exceeding recommended thresholds. The most critical growth was observed for the most severe patients, as mean waiting time for yellow and red admissions peaked at 81 (+100%) and 39 (+290%) minutes (m) in 2024, respectively. The mean process time also substantially increased in post-pandemic years, especially for yellow (from 300 m to 476 m) and red codes (from 330 m to 607 m). The trend was similar for both genders, in all age-classes, and higher in larger hospitals. Multivariable analyses confirmed a significant increase in ED time over the years. Conclusions: These findings indicate critical organizational and clinical issues in the regional emergency care system, requiring immediate action. The Regional Healthcare System recently tried to reduce ED overcrowding with specific plans, the impact of which requires urgent evaluation.

## 1. Introduction

The World Health Organization defines quality healthcare as an effective, safe, and people-centered service delivered in a timely manner while minimizing harm and resource waste [1]. This is especially true for the emergency setting, as delays are associated with worse clinical outcomes, psychological distress, lower patient satisfaction, increased adverse events, medico-legal issues and aggression, and higher healthcare costs [2,3,4,5,6,7,8].

As a consequence, both Emergency Department (ED) waiting time and total time of stay, proxies of timeliness, accessibility, and efficacy and efficiency, are included among the key indicators of ED quality in several quality assessment systems (or by several agencies of quality evaluation) [9,10,11,12,13].

In Italy, as well as in other countries, ED access is regulated through a structured triage system that assigns priority according to clinical severity [14,15]. Defined thresholds for waiting times and overall ED length of stay have been set and are currently monitored by the Ministry of Health and Italian National Agency for Regional Health Services (AGENAS) [12,13]. The latest report showed a critical situation in many emergency facilities across the country, with a significant percentage of Emergency Room (ER) visits exceeding the maximum time established by recommended standards [12]. Also, a recent national survey by the Italian Society of Emergency Medicine (SIMEU) highlighted critical issues across Italian EDs, including inadequate specialist staffing, prolonged waiting times, and increasing rates of patients leaving before completing treatment [16].

Among the Italian regions, Abruzzo is particularly affected by these structural vulnerabilities, with prolonged ED waiting times prompting formal investigations and administrative scrutiny of local health authorities, thereby linking emergency care performance to governance accountability [17]. Concurrently, chronic shortages of emergency physicians, especially in peripheral and inland areas, have necessitated temporary staffing arrangements, reflecting a broader national workforce crisis that disproportionately affects acute and critical care services [16,18].

However, in this scenario of systemic pressure, including organizational strain, increasing waiting times, and workforce shortages, no overall quantitative data are available so far on the magnitude, temporal evolution, and underlying drivers of ED performance. This observational study, using the entire healthcare administrative data of the Abruzzo region, aimed to evaluate temporal patterns in ER visit volume, waiting time, and process time and identify determinants associated with prolonged waiting and process times.

## 2. Materials and Methods

### 2.1. Study Design and Data Sources

A retrospective observational analysis was conducted using the following data from the official database of the EDs in the Abruzzo region of Italy: age and gender, triage codes, and the exact time of access, first visit, and discharge. The above information was collected for all the individuals who accessed an ED in one of the 16 hospitals of the Abruzzo region between 1 January 2017 and 31 December 2024, and were provided by the Regional Office of Healthcare Statistics after full anonymization.

The information on the personnel, location and organizational changes was obtained through a dedicated survey, administered to the head nurse of each of the 16 EDs in the region. The survey assessed whether relevant changes occurred between 2017 and 2024 and required respondents to report the exact timing of each modification. Specifically, the questionnaire gathered detailed information on changes in the director, as well as modifications to the organizational model and structural configuration of the ED (the full questionnaire is reported in Supplementary Materials). For each of the three variables, the reference group consisted of the admissions that occurred before the year of the change.

Ethical review and approval were waived because the study protocol was approved by the internal board of the Abruzzo region, which did not request a formal evaluation from the Regional Ethics Committee. Furthermore, the analyses were conducted using only secondary, entirely anonymized and routinely collected data.

### 2.2. Outcomes

Two outcomes were measured: (1) waiting time (in minutes) which is the time elapsed between ED access (patient registration) and the first medical visit; (2) process time (in minutes) which is the time between the first medical visit and the ED discharge/exit.

Both outcomes were computed overall and stratified by triage code [19]:

− White (non-urgent access: not critical, no immediate risk for the patient; treatment can be deferred);
− Green (minor urgency: not highly critical, low risk of condition worsening, treatment can be delayed);
− Yellow (urgency: moderately critical, increased risk of worsening, potential danger of death, and treatment cannot be delayed);
− Red (emergency: life-threatening condition, maximum priority, requiring immediate intervention).

During the study period, a new triage color (blue) was introduced to specifically identify deferrable cases indicating stable condition without elevated clinical risk [19]. Since this code was not used in Italy throughout the entire observational period, blue codes were grouped together with green codes to ensure comparability across years [19].

### 2.3. Data Analysis

The annual number of ED accesses and the distribution of triage codes were summarized using frequencies and percentages. Waiting time and process time were reported as annual mean and median (in minutes) and interquartile range (IQR). Differences in both waiting and process times across study years were assessed using the Kruskal–Wallis test, and multivariable mixed-effects regression models were used to evaluate the potential predictors of both waiting and process time, setting the individual hospital as the cluster variable. All the following extracted variables were included a priori in the models: sex, age, triage code, year of ED access, change in the director, change/update in the facilities, and change in the organizational model. Multicollinearity was examined using the variance inflation factors (VIFs), and values exceeding 10 were considered indicative of significant collinearity [20].

Statistical significance was defined as a two-sided *p*-value < 0.05 for all analyses, which were performed using Stata 13.1 (Stata Corp., College Station, TX, USA, 2017) and R 4.5.2 (R Foundation for Statistical Computing, Vienna, Austria, 2025). The raw dataset of the study is available in the online Supplementary Materials.

## 3. Results

### 3.1. Overall Number of Accesses and Clinical Severity (Table 1)

From 2017 to 2024, a total of 3,563,565 ED accesses were recorded in the 16 EDs of the Abruzzo region. The overall number of accesses per year remained quite stable at approximately 520,000 from 2017 to 2019, dropped to 335,642 during the pandemic year 2020, then rose progressively in the subsequent years, reaching a new peak (n = 450,530) in 2024.

The proportion of accesses due to a severe condition (yellow or red triage codes) varied over the full observation period: the prevalence of red-coded accesses increased from 2.1% in 2017 to 4.5% in 2024, whereas yellow admissions decreased from 40.1%, to 32.3%. Green-coded accesses increased from 53.2% to 59.6%, while the proportion of white-coded accesses decreased over time, reaching 3.4% in 2024.

**Table 1:** Yearly distribution of Emergency Department (ED) accesses and mean waiting and process times (in minutes) stratified by triage code during the observational period (2017–2024).

			Triage Code		
Year	Overall	White	Green	Yellow	Red
Number (%) of ED accesses					
2017	521,000	23,167 (4.4)	277,380 (53.2)	208,823 (40.1)	11,105 (2.1)
2018	524,609	26,111 (5.0)	288,908 (55.1)	198,340 (37.8)	10,914 (2.1)
2019	512,454	24,574 (4.8)	285,091 (55.6)	190,309 (37.1)	12,146 (2.4)
2020	335,642	16,060 (4.8)	174,004 (51.8)	134,485 (40.1)	10,923 (3.3)
2021	379,406	21,234 (5.6)	193,889 (51.1)	152,493 (40.2)	11,585 (3.1)
2022	420,116	22,568 (5.4)	235,143 (56.0)	149,097 (35.5)	13,212 (3.1)
2023	419,808	20,796 (5.0)	248,334 (59.1)	138,413 (33.0)	12,235 (2.9)
2024	450,530	15,120 (3.4)	268,327 (59.6)	145,328 (32.3)	20,399 (4.5)
Waiting time, mean (SD)					
2017	50/19 (53)	42/10 (47)	60/24 (67)	40/17 (41)	10/6 (7)
2018	49/20 (55)	40/8 (42)	57/23 (68)	41/19 (46)	10/6 (8)
2019	53/21 (62)	40/6 (41)	60/23 (72)	47/22 (53)	12/6 (9)
2020	47/17 (50)	31/4 (24)	53/18 (59)	44/19 (47)	11/6 (9)
2021	62/20 (60)	30/4 (18)	65/21 (67)	67/24 (62)	15/7 (9)
2022	76/33 (81)	39/4 (35)	81/40 (90)	79/32 (78)	22/7 (11)
2023	70/32 (82)	38/2 (36)	77/39 (90)	69/31 (77)	13/7 (10)
2024	78/37 (90)	48/13 (49)	81/41 (94)	81/39 (92)	39/9 (40)
Process time, mean (SD)					
2017	183/103 (148)	73/53 (96)	115/80 (121)	270/145 (185)	322/129 (168)
2018	187/106 (152)	73/51 (97)	117/82 (122)	287/156 (196)	310/130 (172)
2019	202/113 (160)	78/56 (99)	126/87 (127)	312/166 (209)	353/144 (198)
2020	216/117 (176)	84/58 (102)	126/85 (127)	325/178 (227)	365/172 (211)
2021	244/124 (185)	98/69 (113)	136/89 (128)	374/190 (253)	469/204 (313)
2022	294/121 (197)	104/63 (115)	158/89 (142)	480/196 (302)	706/252 (555)
2023	319/121 (191)	97/65 (117)	160/91 (138)	557/199 (320)	918/264 (812)
2024	346/122 (198)	267/37 (141)	258/109 (164)	476/160 (260)	607/110 (320)

Waiting time: time from ED access to medical visit. Process time: time from medical visit to ED discharge. IQR: interquartile range. The differences across years in mean and median waiting or process time, and in the proportion of triage codes, are all significant (*p* < 0.05). From 2017 to 2024, 0.1–0.3% of ED accesses lacked a recorded triage code or involved a patient who died during triage.

### 3.2. Change in Waiting and Process Time During the Follow-Up

**Figure 1:**
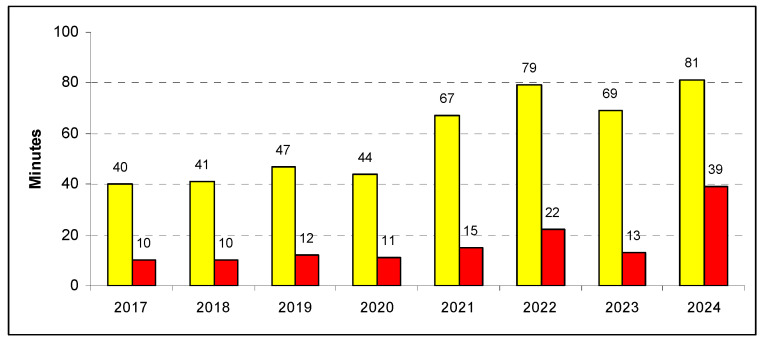
Mean waiting time in the hospitals of Abruzzo, Italy, by triage code (yellow and red).

From 2017 to 2020, the overall waiting time remained quite stable around an average of 50 min, with differences depending on clinical severity: 10 min for red codes; 40 min for yellow and white codes; and 60 min for green accesses. Despite a lower overall number of accesses, from 2021, the waiting time for the first visit steadily increased, reaching an average of 78 min in 2024 (+56.0%). The most critical raise was observed for the most severe patients, as mean waiting time for yellow and red admissions peaked at 81 (+100%) and 39 (+290%) minutes in 2024, respectively (Table 1; Figure 1). Notably, as opposed to the mean, the median waiting time of the red accesses only marginally increased in 2024, and such a discrepancy was explained by the sharp rise in very long waits (≥120 min) from 2023 to 2024 (1.1% vs. 9.7%, respectively).

**Figure 2:**
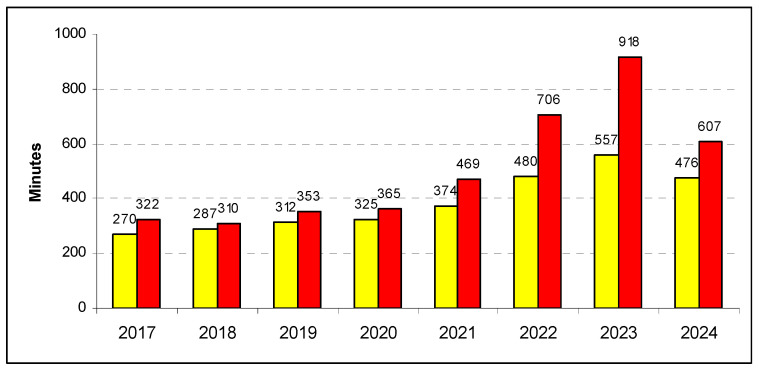
Mean process time in the hospitals of Abruzzo, Italy, by triage code (yellow and red).

Mean process time slightly increased in the first four years, but then it showed a sharp increase from the year 2021, going from approximately 200 min to the peak of 346 min in 2024 (+73%; Table 1). Like the waiting time, the process time was substantially longer for all types of accesses: red codes required an average of approximately 330 min in the first four years and 607 min in 2024 (+84%, with the largest absolute growth of 277 min per patient, Figure 2); yellow codes raised from ~300 to 476 min (+59%); green codes from ~120 to 258 min (+115%); and white-coded admissions from 80 to 267 min (+234%). In contrast with the mean, the median process time of the yellow and red accesses sharply decreased in 2024, with values that were lower than those of the year 2019 (160 and 110 min, respectively). As for the waiting time, this discrepancy is due by a much higher proportion of very long admissions (≥7 days) in 2024 (0.25% of yellow codes and 0.45% of red codes) when compared to the pre-pandemic years (0.01% for both codes).

Notably, the above trend over time in both waiting and process time was observed for both genders, in all age-classes, and in approximately half of the hospitals (Tables S1 and S2). The sharp increase in time at the remaining half of the hospitals started a year later in 2022.

### 3.3. Predictors of ED Waiting/Process Time

Adjusting for sex, age, triage code, hospital size, and occurrence of organizational or structural changes in EDs, multivariate analyses confirmed a significant increase in both waiting time and process time during the post-pandemic (2020–2024) period (Table 2). Compared with the pre-pandemic period (2017–2019), waiting time increased progressively and markedly from 2021 onwards, reaching an excess of 31 min in 2022 and 2024 (*p* < 0.001), and process time raised even more, with a peak of +104 min during 2024.

Regarding other potential independent predictors of waiting/process time (Table 2 and Supplementary Table S3), no relevant differences emerged for sex, whereas an older age was associated with a longer process time. The accesses assigned to white or red triage codes showed an obvious pattern reflecting the clinical complexity, as red accesses are associated with the shortest waiting time and the longest process time.

Notably, both process and waiting times were significantly longer (+42 and +61 min, respectively; both *p* < 0.001) in the EDs of the four largest hospitals in the region (the hub hospitals of the four Local Health Units), as compared to the smaller ones. Both a change in the ED director and a change/upgrade in the facilities did not show any relevant impact, while the process time showed a substantial increase after a change in the director (+49 min; *p* < 0.001) and a change in the organizational model (+54 min; *p* < 0.001), which occurred across both the pre- and post-pandemic periods (Table 2 and Supplementary Table S3).

## 4. Discussion

The official data from the EDs of the Abruzzo region showed a large and progressive increase in both waiting (+56%) and process time (+73%) in the four years following the pandemic (2021–2024). Although there was some heterogeneity across hospitals, the increasing trend affected all triage codes, and the time between ED admission and medical evaluation recorded in 2024 for red and yellow codes largely exceeded the maximum thresholds established by the Italian Ministry of Health (yellow codes: 81 min vs. 15 min recommended by the Ministry; red codes: 39 min vs. immediate access) [19,21].

This trend indicates a critical situation that is consistent with the findings reported by the Italian Agency for Regional Healthcare Services (AGENAS). According to the 2023 data report, 59% of ED visits in Abruzzo met the recommended maximum waiting time for medical evaluation, a value below the national average (67%). Notably, several other Italian regions also reported proportions below the national benchmark, with even worse values reported from Lazio (56%) and Sardinia (53%) [12]. In the same year, AGENAS data also showed that the average length of stay in the regional EDs—including both waiting and process times—exceeded the national average for green (207 min), yellow (407 m) and red (490 m) triage codes [12].

The general increase in waiting and process time observed in the EDs of Abruzzo is corroborated by previous studies conducted in other Italian settings. An observational, retrospective study in the provinces of Milan and Monza (Lombardy) found an overall prolongation of the total ED length of stay during the post-pandemic period, which reached an average time of 8.7 h in 2022 that compared poorly to 2019 (4.9 h) [22]. Similarly, the present study also highlighted an approximate doubling of total length of stay in the EDs, which rose from an average of 233 min in 2017 to 424 min in 2024.

Another single-center retrospective study conducted in the Piedmont region observed a significant prolongation of mean ED length of stay, which doubled during the pandemic and remained stable in 2022 (957 min) compared with the pre-pandemic period (435 min) [23]. Total ED stay time was divided into time to medical evaluation (waiting time) and process time. Waiting time increased from an average of 52 min in the pre-pandemic period to 79 min during the pandemic, and then further to 135 min in the post-pandemic period, which is consistent with the findings of the present study (50 min in 2017–2019, 76 min in 2022). Process time rose from an average of 320 min in the pre-pandemic period to 758 min during the pandemic, before declining to 561 min in the post-pandemic period [23]. Notably, in the Abruzzo cohort, process time progressively increased from an average of 200 min in 2017–2019 to 294 min in 2022 and 346 min in 2024, with peaks exceeding 10 h in some hospitals. A retrospective study including ED data from the Lombardy region also confirmed an increase in average ED length of stay in the post-pandemic period compared with 2019, both for admitted patients (3.8 h in 2019 vs. 5.2 h in 2023) and for discharged patients (2.4 h in 2019 vs. 2.7 h in 2023). Compared to the findings from our analysis, the waiting time was generally shorter in Lombardy, and despite changes during the pandemic it returned to pre-pandemic levels in 2023 (median time 35 min) [24].

Overall, these findings outline a highly critical situation in the regional EDs that has obvious potential implications as delays in clinical evaluation and hospitalization are linked to an increased risk of adverse outcomes, including mortality [25,26], higher levels of patient dissatisfaction [27], overcrowding [28], and potential medico-legal implications for physicians and hospital management [29].

The observed critical scenario requires an in-depth analysis of the underlying reasons in order to urgently implement the appropriate solutions. Some of the potential explanations for the observed trend include an increase in ED admissions and/or decrease in hospital beds, with subsequent ED overcrowding [30], low staffing and resource levels [31], structural and organizational changes [9], and the presence of a considerable proportion of junior medical staff [31].

With regard to ED overcrowding, the overall ED admission did not increase, and the total number of hospital beds did not decrease. In fact, compared with the pre-pandemic period, the total number of ED attendances was substantially lower during the post-pandemic period (520,000 vs. 451,000 in 2024; personal communication). A similar fluctuating trend was observed by previous reports in Italy, which found that total ED visits increased in the post-pandemic era compared to the pandemic period, yet remained below 2019 levels [24,32]; meanwhile, other studies indicated a full return of ED attendance volumes to pre-pandemic levels following the decline observed during the pandemic [14,23]. Concerning the total number of hospital beds where ED patients could be directed, if the occupancy rate increased due to a reduction in the beds and/or increase in hospital admissions, then this might have created a bottleneck and lead to stagnation [30]. However, the available official data indicate that the opposite occurred: the overall number of beds slightly increased, and the total admissions slightly decreased [33].

A second potential explanation for the observed trend in length of stay could be a decrease in the number of ED personnel over time. However, although the overall number of physicians assigned to the ED is generally considered to be below the actual staffing needs, the informal data provided by the Abruzzo region shows that the number of permanently employed physicians in EDs did not decline over the years and the official estimates show a 2.34% increase in National Health Service physicians in the region over the last decade [34].

As quality is inherently multidimensional and also depends on infrastructure, organizational processes, and the ability to respond appropriately to varying levels of clinical acuity [8,9,35], other potential aspects that may influence the emergency settings’ waiting and process time could be structural and organizational changes, such as a change in the ED director, as well as a change/upgrade in the facilities. Although in our multivariable analyses none of them showed a positive impact, the organizational variables in this study were based on a basic survey and the related findings can only be considered as exploratory.

A fourth factor that is known to prolong ED process time is the presence of a considerable proportion of junior medical staff [31]. Indeed, in recent years Italy has experienced a progressive wave of physician retirements, with annual exits estimated at around 11,000–13,000 [36]. This has led to a generational shift within the healthcare workforce of EDs, with newly hired personnel requiring additional time to develop and consolidate clinical experience and professional skills. Therefore, this may have contributed to the critical issues described in the region.

As many of the underlying reasons for the observed rise in ED length of stay remain unexplained, further investigations are urgently needed to identify the main issues, for example, through regional audits involving primarily the directors of the hospitals facing the most critical situations, in order to identify targeted interventions to halt and reverse this upward trend.

A major strength of the present study is the inclusion of the official data ED from all of the 16 EDs across a whole Italian region, spanning an eight-year timeframe. Given that the COVID-19 pandemic profoundly influenced organizational structures and utilization patterns in EDs, the extensive observation period allowed for a comparison of pre-pandemic, pandemic, and post-pandemic phases regarding the entity of ED access and the length of stay. Importantly, our study extends beyond a simple evaluation of total length of stay by providing a more granular analysis of waiting time and process time—dimensions often not analyzed individually in comparable studies.

The study has also some limitations that must be considered. First, potential changes in personnel, location, and organizational aspects were investigated through questionnaires administered to head nurses; therefore, these data were based on subjective reports rather than administrative records. Second, triage code assignment was not independently verified; therefore, misclassification of patients across triage categories cannot be entirely ruled out. Moreover, although unlikely, changes in triage assessment practices over the observation period cannot be completely excluded, which may affect data comparability. Third, official staffing data and some important drivers of ED length of stay, such as admission status, boarding, investigations, consultations, or time-of-day effects, were not available, precluding a more in-depth analysis of factors potentially underlying the observed trends. Finally, about 10% of the ED visits lacked information on sex or age, which can be due to the patient leaving before the first visit (if a patient is not residing in the region) or, infrequently, because of urgent clinical priorities limiting complete documentation. To explore this issue, we required the regional office to perform a linkage of these patients to the regional demographic dataset, and they were able to find only 1.0% of the data. Therefore, unfortunately, 99% of the missing data were of patients that do not reside in the Abruzzo region, and they could not be retrieved.

## 5. Conclusions

The analysis of all ED admissions of the Abruzzo region of Italy over eight years showed that, despite a slight reduction in ED visits throughout the full observation period, both waiting times and process times have increased significantly, reaching their highest values in the post-pandemic period and largely exceeding the maximum thresholds established by the Italian Ministry of Health. This finding suggests an increasing organizational and clinical complexity in emergency care, in which a lower patient volume does not necessarily translate into improved efficiency and productivity. The trend we observed represents a pressing public health concern and calls for urgent interventions to investigate the underlying causes more thoroughly, with the aim of identifying targeted strategies to address the issue, particularly focusing on hospitals exhibiting the worst performance indicators. As the Regional Healthcare System recently tried to reduce ED overcrowding with specific plans including bed managers and other interventions [37], an evaluation of the impact of such measures is urgently needed.

## Figures and Tables

**Table 2:** Multivariate analysis evaluating potential predictors of (a) waiting time (in minutes) or (b) process time in the Emergency Departments (EDs) of the Abruzzo region, Italy.

Variables		Waiting Time			Process Time	
	β	(95% CI)	*p*-Value	β	(95% CI)	*p*-Value
**Male gender**	1	(1; 1)	0.002	6	(5; 8)	<0.001
**Age** (10-year increase)	5	(5; 5)	<0.001	41	(41; 42)	<0.001
**Triage**						
White (reference category)	0	--	--	0	--	--
Green	36	(35; 37)	<0.001	97	(93; 100)	<0.001
Yellow	14	(13; 15)	<0.001	**257**	(254; 261)	<0.001
Red	−40	(−42; −39)	<0.001	**312**	(306; 317)	<0.001
**Year of ED access**						
2017–2019 (reference category)	0	--	--	0	--	--
2020	−2	(−3; −1)	<0.001	5	(3; 8)	<0.001
2021	17	(16; 17)	<0.001	40	(37; 42)	<0.001
2022	31	(30; 32)	<0.001	93	(90; 96)	<0.001
2023	24	(23; 25)	<0.001	99	(95; 102)	<0.001
2024	31	(30; 32)	<0.001	104	(100; 107)	<0.001
Hospital *						
Small hospitals (reference category)	0	--	--	0	--	--
Large hospitals	42	(41; 42)	<0.001	61	(59; 62)	<0.001
**Change in the Director**						
No (reference category)	0	--	--	0	--	--
Yes	6	(5; 7)	<0.001	49	(46; 52)	<0.001
**Change/update in the facilities**						
No (reference category)	0	--	--	0	--	--
Yes	−9	(−9; −8)	<0.001	14	(11; 16)	<0.001
**Change in the organizational model**						
No (reference category)	0	--	--	0	--	--
Yes	2	(1; 2)	<0.001	54	(51; 57)	<0.001

Waiting time: time from ED access to medical visit. Process time: time from medical visit to ED discharge. In both models, no evidence of multicollinearity was detected, as all variance inflation factors (VIFs) were below the accepted threshold of 10. Outcome—waiting time: mixed-effects regression model including 3,035,135 observations. Outcome—process time: mixed-effects regression model including 2,939,054 observations. * This variable was estimated using a different, multiple regression model, in which the hospital of the admission was only adjusted for in the model. It would have been impossible to evaluate this covariate if the hospital was used as a cluster variable in mixed-effect regression, as we did for the rest of the potential predictors presented in the table.

## Data Availability

The raw dataset of the study is available in the online Supplementary Materials.

## Supplementary Materials

The following supporting information can be downloaded at: https://www.mdpi.com/article/10.3390/epidemiologia7030062/s1, Table S1: Mean and median annual waiting time (in minutes) in Emergency Department (ED), stratified by gender, age, and hospital; Table S2: Mean and median annual process time (in minutes) in Emergency Department (ED), stratified by gender, age, and hospital; Table S3: Demographic characteristics of Emergency Department (ED) accesses and organizational and structural changes among EDs of the Abruzzo region, Italy, by period (2017–2020 and 2021–2024); Supplementary Materials: Questionnaire used in the study and Database.
