# Supplementary material for: Continuous Increase in Both Waiting and Process Time in the Emergency Rooms of Abruzzo, Italy
Source: Epidemiologia (Basel). 2026 May 4;7(3):62. doi: 10.3390/epidemiologia7030062 (PMC13214812; doi:10.3390/epidemiologia7030062)
Supplement: Supplementary file 1 [file epidemiologia-07-00062-s001.zip › Supplementary Materials.pdf]

Supplementary Materials- Questionnaire

1. Between 01/01/2017 and 31/12/2024, were the Emergency Department facilities changed or modified (e.g., relocation, major structural renovation)?
□ No
□ Yes

If yes, please indicate the date: ___________

2. Between 01/01/2017 and 31/12/2024, did the Emergency Department undergo a substantial organizational model change (excluding temporary changes implemented during the acute phases of the COVID-19 pandemic)?
□ No
□ Yes

If yes, please indicate the date: ___________

3. Between 01/01/2017 and 31/12/2024, was there a change of the ED director (or Acting director)?
□ No
□ Yes

If yes, please indicate the date: __________
