# Supplementary material for: Continuous Increase in Both Waiting and Process Time in the Emergency Rooms of Abruzzo, Italy
Source: Epidemiologia (Basel). 2026 May 4;7(3):62. doi: 10.3390/epidemiologia7030062 (PMC13214812; doi:10.3390/epidemiologia7030062)
Supplement: Supplementary file 1 [file epidemiologia-07-00062-s001.zip › Tables S1-S3 revised.pdf]

Table S1. Mean and median annual waiting time (in minutes) in Emergency Department (ED), stratified by gender, age, and hospital.

	2017	2018	2019	2020	2021	2022	2023	2024
**Gender**								
Female	50 / 19 (54)	50 / 20 (58)	55 / 21 (63)	47 / 16 (51)	63 / 19 (62)	76 / 33 (82)	71 / 33 (84)	79 / 39 (91)
Male	49 / 19 (51)	48 / 20 (54)	51 / 21 (59)	47 / 18 (49)	62 / 21 (60)	76 / 33 (80)	69 / 31 (79)	77 / 36 (89)
**Age**								
<18	29 / 9 (27)	28 / 9 (30)	28 / 10 (31)	25 / 10 (26)	35 / 12 (31)	46 / 24 (50)	40 / 18 (45)	41 / 22 (47)
>=18-64	54 / 21 (59)	54 / 22 (63)	59 / 23 (69)	50 / 17 (55)	64 / 20 (65)	78 / 34 (88)	75 / 35 (90)	85 / 43 (101)
>=65-74	53 / 21 (54)	51 / 22 (57)	56 / 24 (65)	49 / 19 (53)	68 / 24 (67)	86 / 36 (91)	79 / 37 (94)	86 / 42 (103)
>=75-84	51 / 21 (52)	50 / 23 (57)	55 / 24 (63)	49 / 20 (52)	71 / 25 (69)	88 / 37 (92)	80 / 39 (94)	89 / 44 (109)
>=85	47 / 20 (49)	47 / 22 (52)	50 / 23 (57)	46 / 20 (48)	69 / 24 (65)	87 / 35 (88)	77 / 37 (90)	84 / 39 (99)
**Hospital**								
1 (Hub)	45 / 20 (47)	46 / 20 (47)	48 / 20 (53)	45 / 16 (46)	56 / 21 (61)	89 / 47 (93)	81 / 38 (93)	78 / 36 (91)
2	25 / 11 (20)	23 / 10 (18)	19 / 9 (16)	13 / 6 (10)	23 / 8 (16)	27 / 11 (21)	29 / 13 (27)	34 / 18 (35)
3	22 / 12 (25)	22 / 13 (26)	22 / 12 (26)	20 / 10 (23)	22 / 11 (24)	29 / 17 (34)	35 / 22 (39)	47 / 26 (51)
4	18 / 8 (17)	20 / 9 (18)	22 / 9 (19)	21 / 9 (17)	25 / 13 (20)	40 / 29 (28)	32 / 17 (32)	31 / 15 (27)
5	48 / 9 (20)	25 / 11 (26)	25 / 10 (25)	17 / 8 (15)	22 / 10 (21)	27 / 12 (27)	31 / 13 (32)	44 / 21 (50)
6	30 / 12 (33)	33 / 14 (36)	32 / 15 (37)	27 / 12 (28)	30 / 14 (31)	46 / 23 (50)	53 / 30 (59)	52 / 29 (62)
7	37 / 16 (41)	34 / 15 (36)	35 / 14 (35)	35 / 15 (36)	38 / 15 (38)	68 / 37 (81)	57 / 28 (61)	49 / 24 (52)
8	26 / 10 (29)	30 / 13 (33)	37 / 16 (41)	41 / 20 (44)	96 / 19 (34)	50 / 31 (45)	46 / 24 (49)	48 / 24 (52)
9	22 / 9 (22)	25 / 9 (26)	32 / 11 (32)	31 / 13 (29)	46 / 18 (49)	57 / 25 (67)	61 / 19 (77)	75 / 28 (94)
10	36 / 15 (35)	34 / 16 (36)	44 / 18 (49)	38 / 15 (38)	50 / 20 (52)	78 / 41 (92)	64 / 37 (73)	76 / 43 (92)
11	40 / 18 (44)	40 / 20 (46)	43 / 23 (51)	36 / 17 (41)	39 / 18 (42)	40 / 17 (45)	53 / 24 (66)	75 / 41 (93)
12	38 / 15 (44)	44 / 17 (53)	50 / 18 (64)	40 / 14 (43)	45 / 15 (49)	66 / 32 (79)	82 / 42 (99)	75 / 37 (89)
13 (Hub)	64 / 19 (76)	59 / 22 (76)	62 / 22 (82)	51 / 17 (57)	47 / 17 (50)	71 / 37 (79)	73 / 40 (83)	77 / 40 (90)
14 (Hub)	47 / 23 (64)	50 / 24 (69)	59 / 27 (85)	46 / 16 (61)	62 / 17 (84)	77 / 20 (111)	74 / 16 (116)	89 / 52 (117)
15	52 / 18 (59)	54 / 21 (67)	66 / 26 (82)	53 / 20 (62)	105 / 28 (83)	93 / 56 (86)	81 / 47 (88)	81 / 46 (87)
16 (Hub)	94 / 51 (89)	93 / 55 (94)	93 / 50 (93)	84 / 41 (96)	115 / 53 (132)	141 / 61 (156)	107 / 58 (129)	121 / 61 (169)

Waiting time: time from ED access to medical visit. All values are reported as mean/median (interquartile [IQR]).

The mean waiting time showed significant differences across years in all the reported categories of gender, age, and hospital (Kruskal-Wallis $p<0.05$).

Table S2. Mean and median annual process time (in minutes) in Emergency Department (ED), stratified by gender, age, and hospital.

	2017	2018	2019	2020	2021	2022	2023	2024
**Gender**								
Female	188 / 107 (408)	192 / 109 (417)	207 / 116 (451)	216 / 118 (481)	250 / 125 (535)	301 / 122 (773)	330 / 122 (896)	359 / 122 (1007)
Male	182 / 101 (393)	185 / 105 (380)	201 / 111 (432)	222 / 118 (536)	248 / 126 (520)	297 / 123 (737)	318 / 121 (844)	348 / 123 (935)
**Age**								
<18	101 / 68 (110)	108 / 74 (116)	116 / 80 (121)	116 / 81 (125)	131 / 97 (137)	139 / 96 (142)	142 / 96 (134)	145 / 92 (134)
>=18-64	148 / 94 (135)	151 / 95 (140)	163 / 102 (148)	166 / 101 (153)	175 / 104 (154)	192 / 101 (163)	200 / 103 (162)	219 / 104 (171)
>=65-74	213 / 118 (162)	220 / 122 (170)	236 / 129 (179)	262 / 138 (198)	290 / 149 (216)	351 / 147 (238)	375 / 147 (234)	413 / 147 (241)
>=75-84	263 / 135 (182)	270 / 140 (188)	295 / 149 (202)	326 / 165 (231)	411 / 186 (278)	534 / 189 (346)	631 / 189 (365)	660 / 190 (371)
>=85	308 / 149 (189)	316 / 154 (197)	350 / 166 (215)	388 / 194 (255)	526 / 229 (369)	755 / 254 (608)	915 / 252 (746)	976 / 256 (800)
**Hospital**								
1 (Hub)	155 / 104 (140)	174 / 112 (149)	185 / 116 (156)	190 / 123 (171)	177 / 136 (170)	254 / 136 (215)	272 / 141 (216)	275 / 128 (203)
2	116 / 41 (121)	84 / 48 (112)	84 / 52 (108)	67 / 34 (87)	83 / 56 (102)	88 / 56 (106)	87 / 64 (106)	104 / 73 (127)
3	113 / 89 (102)	97 / 80 (109)	102 / 80 (110)	118 / 80 (134)	119 / 83 (128)	138 / 84 (120)	164 / 89 (119)	202 / 95 (131)
4	210 / 119 (141)	196 / 115 (138)	183 / 109 (127)	214 / 105 (135)	200 / 100 (136)	252 / 108 (139)	250 / 113 (143)	270 / 127 (158)
5	93 / 62 (101)	101 / 72 (104)	109 / 78 (110)	109 / 75 (103)	151 / 73 (104)	172 / 76 (113)	198 / 81 (128)	198 / 85 (144)
6	99 / 87 (125)	112 / 94 (135)	129 / 108 (136)	152 / 113 (154)	158 / 122 (160)	162 / 117 (153)	150 / 107 (145)	138 / 106 (133)
7	137 / 86 (116)	136 / 89 (117)	139 / 91 (117)	181 / 100 (138)	211 / 104 (150)	228 / 104 (174)	223 / 108 (165)	238 / 111 (164)
8	171 / 94 (127)	178 / 98 (133)	193 / 104 (145)	260 / 120 (172)	261 / 142 (187)	267 / 118 (184)	255 / 118 (186)	250 / 122 (179)
9	262 / 126 (190)	264 / 132 (197)	270 / 144 (212)	263 / 138 (196)	385 / 147 (224)	418 / 145 (218)	474 / 148 (231)	564 / 153 (290)
10	139 / 106 (137)	140 / 106 (139)	160 / 119 (160)	153 / 109 (147)	169 / 113 (148)	189 / 104 (161)	202 / 110 (153)	222 / 114 (156)
11	201 / 125 (181)	219 / 130 (182)	235 / 130 (183)	194 / 122 (166)	193 / 120 (158)	225 / 119 (159)	240 / 123 (164)	257 / 130 (184)
12	130 / 87 (127)	130 / 87 (123)	139 / 91 (128)	160 / 97 (141)	185 / 116 (156)	158 / 98 (150)	150 / 92 (132)	183 / 100 (136)
13 (Hub)	212 / 111 (140)	210 / 111 (143)	213 / 110 (149)	187 / 110 (149)	183 / 110 (142)	193 / 105 (152)	191 / 103 (145)	218 / 110 (153)
14 (Hub)	260 / 126 (140)	266 / 132 (147)	291 / 140 (156)	323 / 144 (166)	392 / 169 (222)	402 / 183 (248)	412 / 174 (235)	411 / 153 (248)
15	250 / 107 (155)	265 / 112 (172)	356 / 131 (210)	338 / 136 (212)	349 / 154 (258)	405 / 154 (283)	474 / 162 (328)	582 / 148 (331)
16 (Hub)	143 / 93 (179)	150 / 97 (191)	160 / 107 (201)	206 / 129 (261)	269 / 128 (286)	491 / 142 (357)	572 / 138 (337)	604 / 140 (316)

Process time: time from medical visit to ED discharge. All values are reported as All values are reported as mean/median (interquartile [IQR]).
Mean process time showed significant differences across years in all the reported categories of gender, age, and hospital (Kruskal-Wallis $p<0.05$).

Table S3. Demographic characteristics of Emergency Department (ED) accesses and organizational and structural changes among EDs of the Abruzzo Region, Italy, by period (2017-2020 and 2021-2024).

	2017-2020	2021-2024
**Demographic characteristics of ED access**		
**Gender, n (%)**		
Male	845,373 (44.6)	754,885 (45.2)
Female	841,603 (44.4)	742,383 (44.5)
Missing	206,729 (10.9)	172,592 (10.3)
**Age, n (%)**		
<18	207,201 (10.9)	223,207 (13.4)
>=18-64	867,972 (45.8)	774,605 (46.4)
>=65-74	208,888 (11.0)	188,515 (11.3)
>=75-84	219,038 (11.6)	186,037 (11.1)
>=85	183,877 (9.7)	124,904 (7.5)
Missing	206,729 (10.9)	172,592 (10.3)
**Organizational and structural changes** [A]		
Change/update of the facilities, n (%)	7 (43.8)	6 (37.5)
Change of the Director, n (%)	4 (25.0)	10 (62.5)
Change of the organizational model, n (%)	2 (12.5)	8 (50.0)

[A]: Organizational and structural changes occurred during the study period were assessed among the 16 included EDs.
The differences across years in sex distribution and age were all statistically significant ($p<0.05$).
